# Editorial: State‐of‐the‐art hypothesis-driven systems pharmacology and artificial intelligence approaches to decipher disease complexity

**DOI:** 10.3389/fphar.2025.1593164

**Published:** 2025-03-26

**Authors:** Cristina Correia, Choong-Yong Ung, Hu Li, James C. Costello

**Affiliations:** ^1^ Department of Molecular Pharmacology and Experimental Therapeutics, Center for Individualized Medicine, Mayo Clinic, Rochester, MN, United States; ^2^ Department of Pharmacology, University of Colorado Anschutz Medical Campus, Aurora, CO, United States; ^3^ University of Colorado Cancer Center, University of Colorado Anschutz Medical Campus, Aurora, CO, United States

**Keywords:** systems biology, artificial intelligence, machine learning, hypothesis testing, complex diseases

Most human diseases are inherently complex, multi-factorial, and often involve intricate interactions between genes, as well as other -omic, external, and phenotypic factors that drive their pathological outcomes (Ung et al., 2022; Ung et al., 2023). Due to the complex etiology of many diseases, the penetrance of most individual disease-associated genes is typically limited and can vary significantly, depending not only on the genetic makeup of individuals but also on their cellular contexts (Castro-Giner et al., 2015). Therefore, there is an urgent need to devise innovative state-of-the-art systems biology and Artificial Intelligence (AI) tools that can unravel the mechanisms and drivers of disease, leveraging the advancement of high-throughput sequencing technologies.

This Research Topic aims to foster open discussion and inspire research interest in developing novel hypotheses that enhance our mechanistic understanding of disease etiology. It also seeks to promote the design of hypothesis-driven systems biology or AI algorithms, tools, models, and resources. Combined, these strategies can promote a systems-based paradigm in pharmacological sciences aimed at advancing individualized and precision medicine (Xianyu et al., 2024).

Goble et al. set out to develop a pipeline to detect and quantify extrachromosomal DNA (ecDNA) as a measure of the adaptive response of cancer to a drug. In contrast, to conventional protein-mediated modifications and genetic mutations, ecDNA can be used as a rapid, reversible, and predictable DNA alteration critical for cancer’s adaptive response. Their methodology, Microscopy Image Analyzer (MIA), leverages imaging data collected from Fluorescence *In Situ* Hybridization (FISH), a gold standard imaging platform for ecDNA detection, with Convolutional Neural Networks (CNN), a power technique for image segmentation, to build a novel post-processing automation pipeline for ecDNA detection. The autodetection of ecDNA in metaphase FISH images remains a challenging task due to high levels of noise and considerable cell-to-cell variation. These factors complicate image processing and signal detection, making accurate analysis difficult. The proposed automated pipeline, MIA, is an advance from traditional, labor-intensive karyotyping methods. The authors successfully applied their methodology to quantify alterations in ecDNA counts before and after drug treatment (JQ1) across four distinct cancer cell lines: a lung cancer cell line (NCI-H2170), a gastric cancer cell line (SNU16), and colorectal cancer cell lines (NCI-H716, COLO320DM). MIA offers a robust approach to quantify ecDNA and provides a means to enhance our understanding of how cancer cells adapt to therapeutic pressures. This approach yields valuable insights for drug discovery and the development of cancer treatment strategies.

Mathematical models are indispensable tools for gaining a mechanistic understanding of complex disease and therapeutic responses. These models allow researchers to quantify and simulate biological processes that can contribute to disease progression and can be particularly valuable in assessing the efficacy of various pharmacological agents within treatment strategies. Thomas et al. devised a mathematical model using a hypothesis-driven systems pharmacology approach to interrogate the long-lasting effect of high glucose levels in glomerular fibrosis and model the drug action in this disease context. Glomerular fibrosis refers to tissue damage, leading to scarring and thickening of the glomeruli in the kidneys. This condition commonly occurs in patients with diabetic kidney disease (DKD), where effective treatments are currently lacking. The progressive kidney damage in DKD is mediated by host-mediated stimuli, such as advanced glycation end products (AGE), hypertension, angiotensin, and enhanced oxidative stress. These factors contribute to glomerular fibrosis and further exacerbate kidney dysfunction. To address the current clinical challenges, the authors developed a mechanistic mathematical model of diabetes-induced kidney damage by adapting a previously established model on interstitial fibrosis in lupus (Hao et al., 2014). This model was tailored to glomerular fibrosis using a system of ordinary differential equations and focuses on relevant kidney cell populations to model the complexity of the disease and progression of glomerular fibrosis in diabetes. Their model recapitulated what has been observed in the clinic. There is a lag phase in the recovery of damage caused by high blood glucose levels, where good blood glucose control does not lead to immediate recovery from glomerular fibrosis in the context of diabetes. Interestingly, their model also predicted that inhibition of AGE production is not an effective approach for accelerating the recovery from glomerular fibrosis, possibly due to the slow breakdown of these species. Another model prediction suggests that a therapeutic approach that enhances AGE degradation could theoretically be highly effective in reversing glomerular fibrosis. Importantly, the model provided important insights into why glucose control and aminoguanidine treatment are ineffective in the short term for reversing advanced glomerular fibrosis.

Orman et al. delved into the complexity of molecular landscapes by extracting multigene co-alteration patterns associated with the aggressiveness of prostate cancer (PCa). The goal of this work was to uncover crucial biological signals that underlie the mechanism of disease progression. The authors, developed ProstaMine, a system biology and integrative genomic tool designed to systematically identify co-alterations of genes that are associated with aggressiveness in PCa. This tool focused on molecular subtypes of PCa, defined by high-fidelity alterations in primary PCa enabling deeper understanding of genetic alterations driving disease progression. ProstaMine leverages multi-omics (genomic, transcriptomic) along with clinical data from five primary and one metastatic PCa cohort, to prioritize co-alterations that are enriched in metastatic disease. ProstaMine was applied to PCa subtypes with NKX3-1-loss and RB1-loss and successfully identified co-altered genes that function in canonical PCa signaling pathways such as MAPK, NF-kB, p53, SMAD, and PI3K. Notably, ProstaMine identified novel subtype-specific co-alterations in p53, STAT6, and MHC class I antigen presentation associated with aggressiveness in RB1-loss PCa. By combining five independent molecular profiling studies in primary PCa, the authors showed that ProstaMine can systematically identify high-confidence alteration hotspots across primary PCa genomes and can detect clinicopathologic features related to aggressiveness. Importantly, the authors showed the ability of ProstaMine to mine any user-selected molecular subtype. ProstaMine can provide insights into subtype-specific mechanisms of PCa progression and aggressiveness, which can be further formulated into testable experimental hypotheses for further clinical discovery and therapeutic strategies.

Finally, array-based platforms are capable of interrogating hundreds of thousands of loci across the human genome in a cost-effective and large-scale manner, spurring genetic studies in the areas such as pharmacogenomics, nutrigenetics, and in complex disease risk prediction. Establishing effective clinical reporting workflows requires comprehensive validation studies to accurately assess assay performance. Accordingly, Gan et al. conducted a systematic study aimed at the development and validation of a clinical reporting workflow for pharmacogenomics testing. The workflow utilizes the Illumina Global Screening Array (GSA) chip, a widely available and cost-effective solution for genetic testing. Their work focuses on 503 distinct genetic variants across 25 pharmacogenomic-related (PGx) genes associated with 303 clinically actionable drugs. This study reports accuracy and precision assessments of their GSA-based platform using well-established reference materials. This information is not available from previous studies and was critical to define robustness of the Illumina GSA chip in preemptive pharmacogenomics testing. The authors presented their results of accuracy and precision assessments across a total of 73 cell lines. Their evaluations also included reference materials such as the Genome-In-A-Bottle (GIAB), the Genetic Testing Reference Material Coordination Program (GeT-RM) projects, and additional samples from the 1000 Genomes project (1KGP). In addition, the authors also conducted an accuracy assessment of genotype calls for target loci in each indication against established truth sets. In their per-sample analysis, they observed a mean analytical sensitivity of 99.39% and specificity 99.98%. Further, they assessed the accuracy of star-allele calls by relying on established diplotypes in the GeT-RM catalogue or calls made based on 1KGP genotyping. On average, the authors detected a diplotype concordance rate of 96.47% across 14 pharmacogenomic-related genes with a set of selected alleles calls (star allele-calls). Lastly, the authors evaluated the reproducibility of their findings across replicates and observed 99.48% diplotype and 100% phenotype inter-run concordance, demonstrating the robustness and reliability of the developed workflow.

Overall, works published in this Research Topic highlight various challenges that have inspired the development of AI and systems biology tools that offer new clinical insights for hypothesis formulation and testing. These works do not mark the conclusion of this Research Topic; rather, they serve as a foundation for future exploration and development. For example, questions remain about how to integrate generative AI and large language models (LLMs) into systems biology tools, and advanced multi-omics data analyses and integration. This would enable hypothesis formulation and help uncover multi-factorial factors that underly disease etiology and progression. We believe this vision will ignite the next wave of interest in AI-powered systems biology research.
